# Supplementary material for: Mussel Mass Mortality and the Microbiome: Evidence for Shifts in the Bacterial Microbiome of a Declining Freshwater Bivalve
Source: Microorganisms. 2021 Sep 17;9(9):1976. doi: 10.3390/microorganisms9091976 (PMC8471132; doi:10.3390/microorganisms9091976)
Supplement: Supplementary file 1 [file microorganisms-09-01976-s001.zip › microorganisms-1369080-SI.pdf]

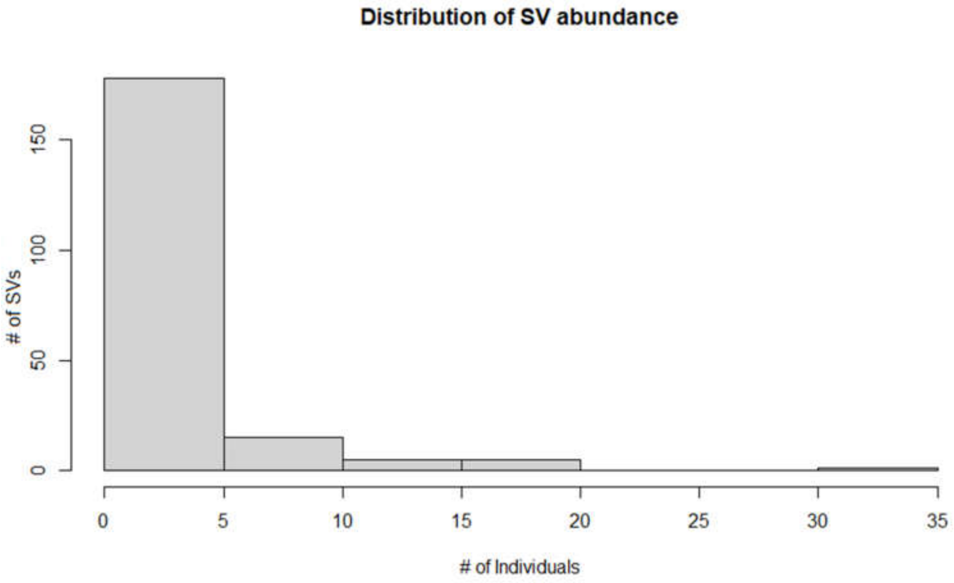

Figure S1. Histogram of SV abundance distribution among all mussel hemolymph samples. The majority of SVs were isolated from relatively few individuals, while only a few SVs were isolated from > 5 individuals.

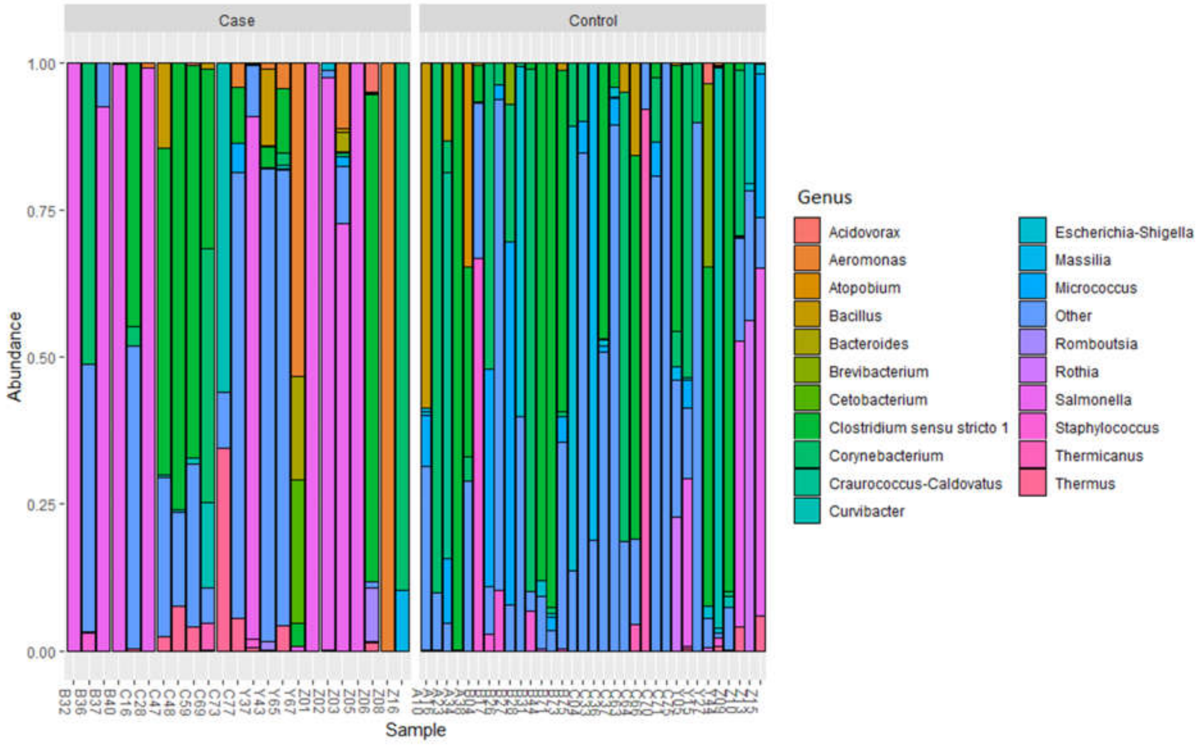

Figure S2. Relative abundance of top 20 bacterial genera in the hemolymph microbiome of moribund mussels (Cases) and apparently healthy mussels (Controls) collected in 2017 and 2018 from 6 sites on the

Clinch River, Virginia and Tennessee, USA, as determined by 16SrRNA gene sequence counts. All SVs attributed to genera outside the top 20 are collectively included in the above plot as “Other”.

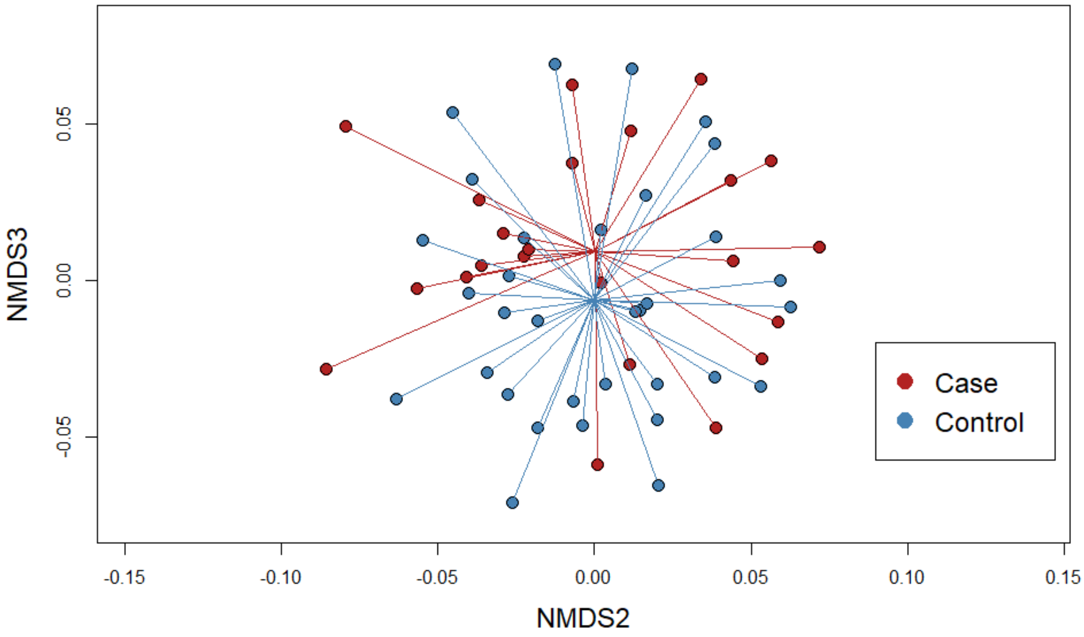

Figure S3. NMDS plot of microbiome ordination colored by clinical status. Plot depicts NMDS axis 2(x) and axis 3(y) and shows no apparent differences between groups based on these axes.

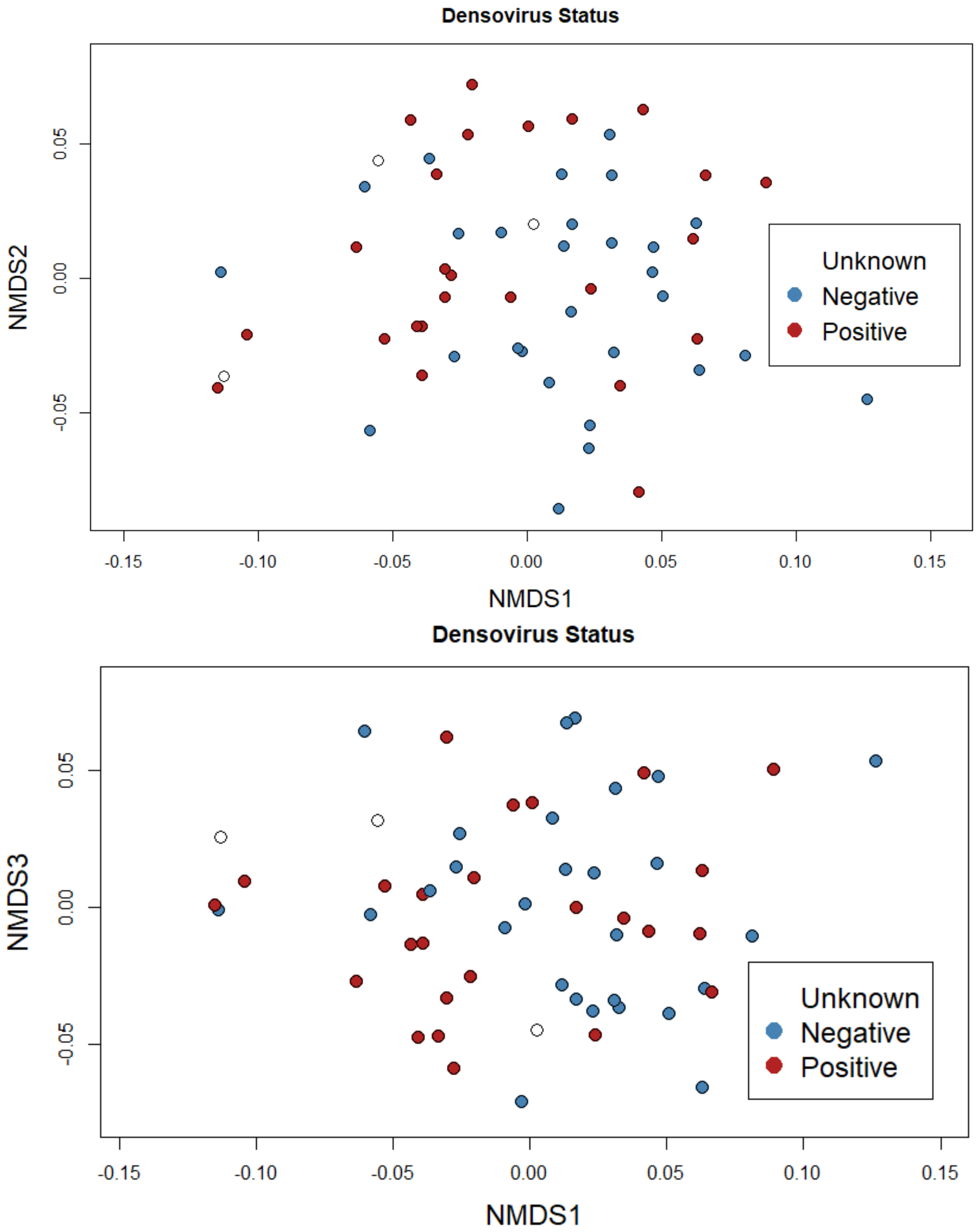

Figure S4. NMDS plots of microbiome ordinations colored by densovirus status. Plots show ordinations based on NMDS axis 1(x) with NMDS axis 2(y) (top) and NMDS axis 2(x) with NMDS axis 3(y) (bottom). Unknown samples are from three individuals included in this study that were not screened for virology in a previous study.

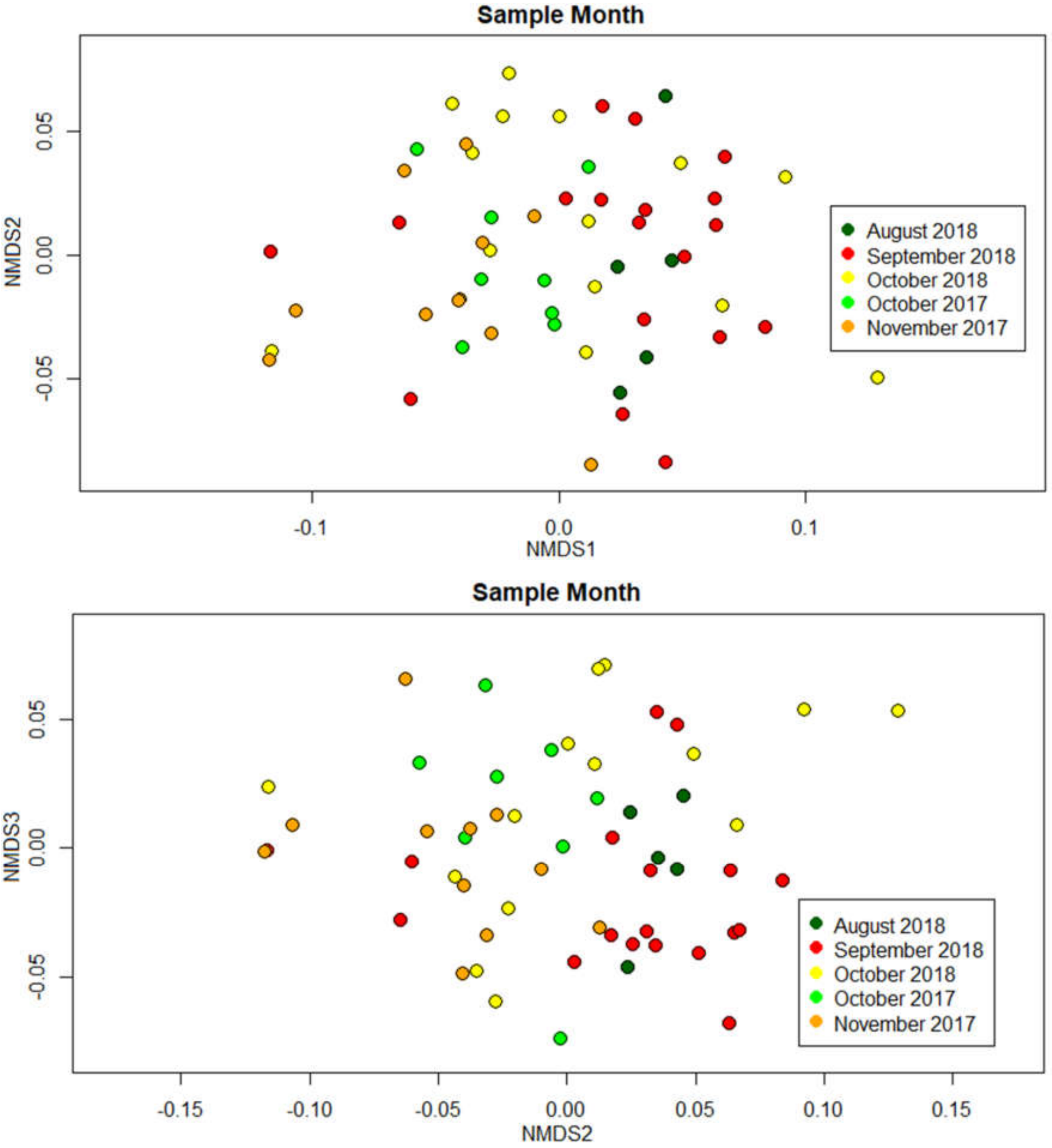

Figure S5. NMDS plots of microbiome ordinations colored by sampling event. Plots show ordinations based on NMDS axis 1(x) with NMDS axis 2(y) (top) and NMDS axis 2(x) with NMDS axis 3(y) (bottom).

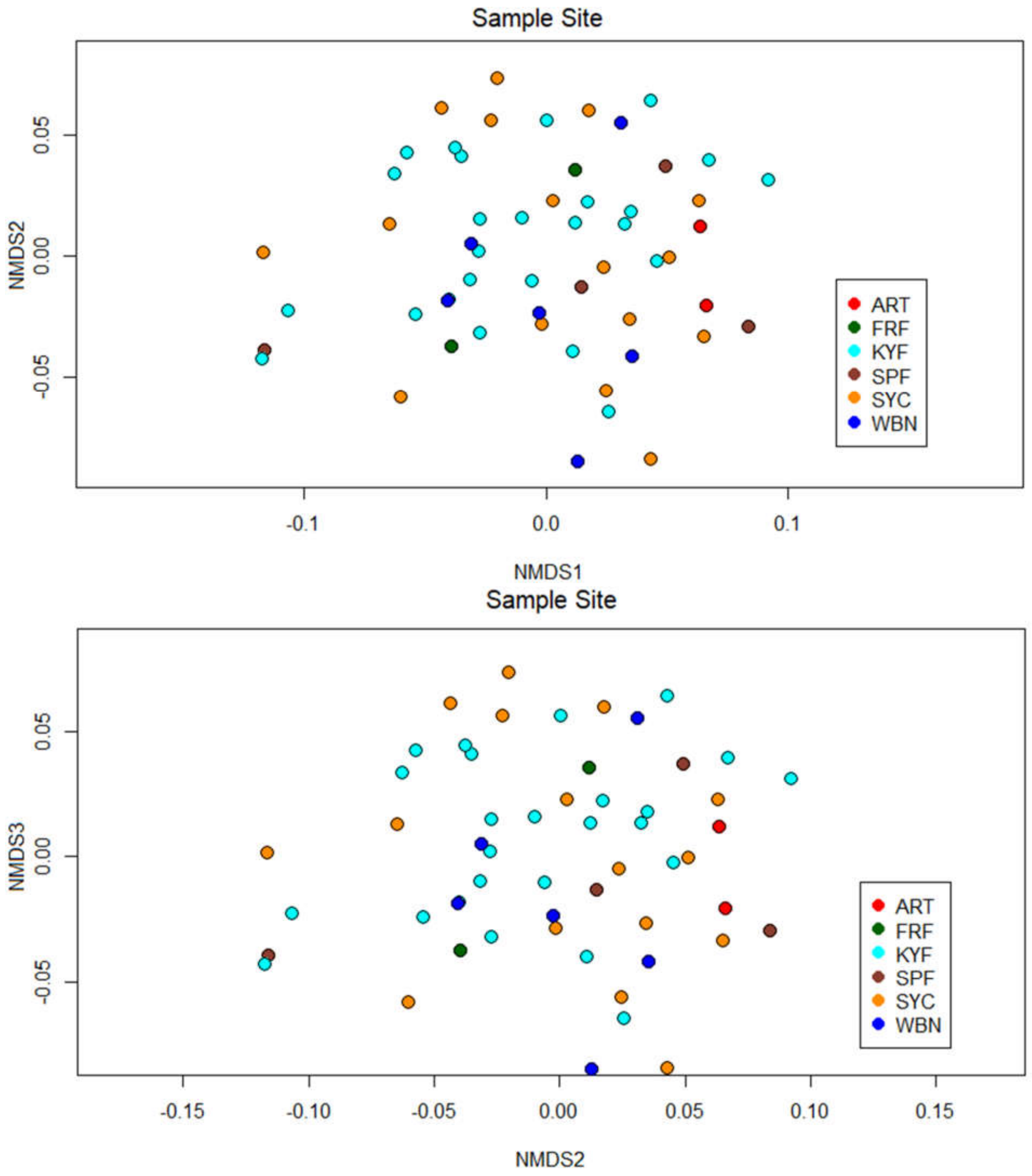

Figure S6. NMDS plots of microbiome ordinations colored by sampling site. Plots show ordinations based on NMDS axis 1(x) with NMDS axis 2(y) (top) and NMDS axis 2(x) with NMDS axis 3(y) (bottom).

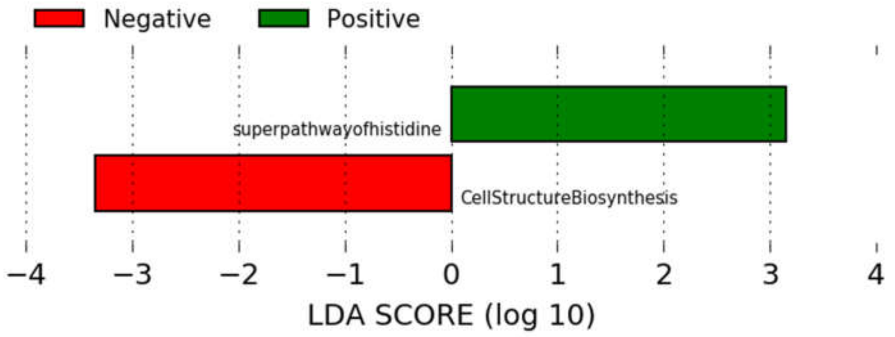

Figure S7. LEfSe plot of inferred mussel hemolymph microbiome function summarized at the second highest MetaCyc pathway classification level.

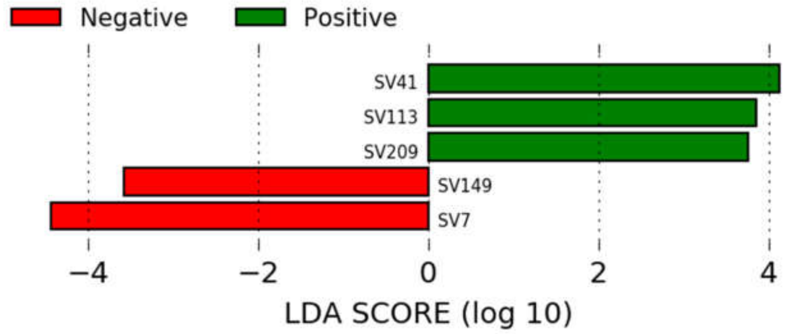

Figure S8. LEfSe plot of SVs significantly associated with mussel microbiomes based on densovirus status.

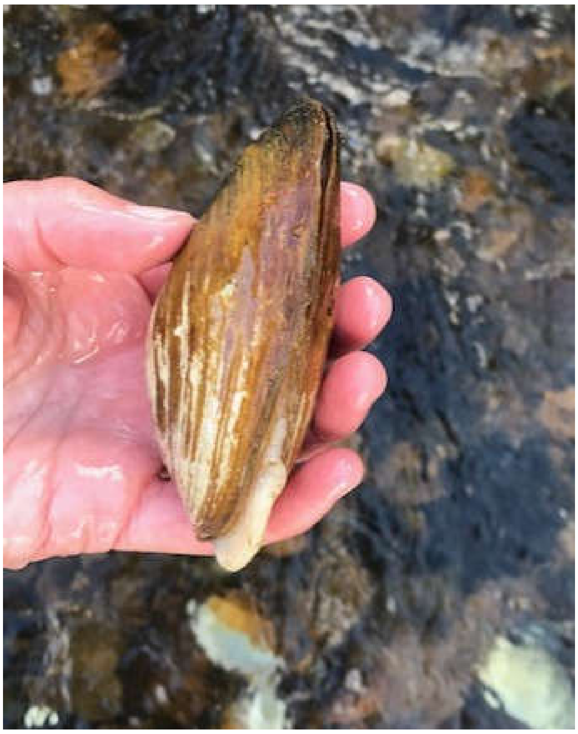

Figure S9. Example of a moribund, gaping pheasantshell collected from the Clinch River. Moribund individuals were differentiated from live, apparently healthy individuals based on their position (generally laying on the substrate surface rather than buried) and their slow, weak responses to tactile stimulation. Note that this figure is provided as an example and was not part of the current study. All samples were handled with gloves during the study.
